# Hierarchical Control Strategy for the Cooperative Braking System of Electric Vehicle

**DOI:** 10.1155/2015/584075

**Published:** 2015-07-05

**Authors:** Jiankun Peng, Hongwen He, Wei Liu, Hongqiang Guo

**Affiliations:** ^1^National Engineering Laboratory for Electric Vehicles, Beijing Institute of Technology, Beijing 100081, China; ^2^School of Mechanical and Automotive Engineering, Liaocheng University, Liaocheng Shandong 252000, China

## Abstract

This paper provides a hierarchical control strategy for cooperative braking system of an electric vehicle with separated driven axles. Two layers are defined: the top layer is used to optimize the braking stability based on two sliding mode control strategies, namely, the interaxle control mode and signal-axle control strategies; the interaxle control strategy generates the ideal braking force distribution in general braking condition, and the single-axle control strategy can ensure braking safety in emergency braking condition; the bottom layer is used to maximize the regenerative braking energy recovery efficiency with a reallocated braking torque strategy; the reallocated braking torque strategy can recovery braking energy as much as possible in the premise of meeting battery charging power. The simulation results show that the proposed hierarchical control strategy is reasonable and can adapt to different typical road surfaces and load cases; the vehicle braking stability and safety can be guaranteed; furthermore, the regenerative braking energy recovery efficiency can be improved.

## 1. Introduction

Cooperative braking is one of the core technologies in electric vehicles [1]. Two advantages can be provided by a good cooperative braking system. First, improved fuel economy can be achieved as a result of energy recuperation when braking [2]. Then, good braking stability can be obtained by a reliable braking force control strategy [3]. Many investigations have been reported for cooperative braking system. General braking condition and emergency braking condition are mainly studied [3–5].

For general braking condition, the peak road adhesion coefficient is high and the wheels will not be locked; the braking strategy focuses on the braking force distribution between front-rear wheels to keep a better braking stability and improve the regenerative braking energy recovery efficiency. For instance, considering the maximum regenerative braking capacity, the road adhesion condition and the braking requirement [4] made an optimal braking control chart for braking force distribution, which can be conveniently applied to engineering. Reference [5] designed a braking force distribution strategy in which the front wheel friction braking force would be adjusted and the rear wheel braking force is only dependent on the braking pedal travel. Nevertheless, for the control strategies of [4, 5], some key parameters should be precisely estimated, which is not easy work. Reference [6] provided a braking force distribution strategy based on the ideal braking force distribution curve (I curve), by which the optimum braking stability was obtained. However, the ideal braking force curve will change as the variation of the loaded condition changes the mass center of the vehicle. The vehicle may fall into braking instability state if the braking strategy could not adapt to the variation of the ideal braking force curve.

For emergency braking cases, the objective is to avoid the vehicle being locked and improve the regenerative braking energy recovery efficiency meanwhile. The main control strategies can be classified into two types. One is based on the logic-threshold control and the other is based on sliding mode control. Reference [7] has proposed an optimized logic-threshold control strategy to improve the cooperative braking performance. References [8–12] provided fuzzy control strategies to improve the vehicle stability. References [3, 13–16] have made some sliding mode control strategies to optimize the regenerative braking stability. Reference [17] has verified that combination of ABS and regenerative braking system can improve barking stability. All of the control strategies have been verified through simulation or road testing and made great efforts to the engineering application. For all the control strategies of the emergency braking condition, a reliable and good robustness control strategy which can adapt to various emergency braking cases is the key technology [18].

In this paper, a hierarchical control strategy which can meet the requirements of the general and emergency braking conditions is proposed. The hierarchical control strategy is designed to have top and bottom layers. The optimum braking stability is the main objective in the top layer. Considering the variation of the loaded condition, an interaxle sliding mode strategy is proposed, based on which the ideal braking force distribution can be realized. Furthermore, to avoid the braking instability in emergency braking condition, a single-axle sliding mode strategy is also designed to meet various emergency cases. The switching condition between the control strategies is based on the threshold value of slip rate. In the bottom layer, the required braking torque will be reallocated between generators and hydraulic brakes; the optimum regenerative braking energy recovery efficiency is the main objective in this layer.

## 2. The Cooperative Braking System Structure

**Figure 1:**
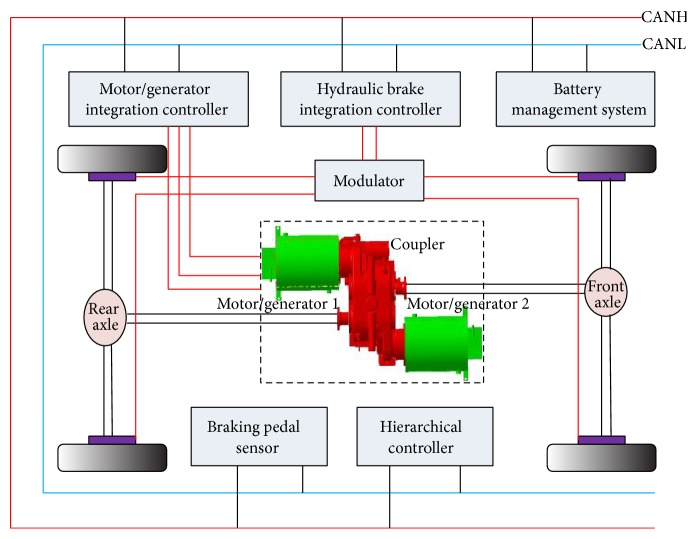
The cooperative braking system structure.

As shown in Figure 1, a coupler together with two motors/generators is equipped in the electric vehicle, based on which four-wheel drive mode can be realized. Additionally, in order to realize the real-time braking torque distribution of the hydraulic brakes, a modulator is also applied in the cooperative braking system. Particularly worth mentioning is that the communication of the vehicle is through the CAN bus. During regenerative braking process, motors are used as generators. Generator 1 provides regenerative braking torque to the front axle; generator 2 provides regenerative braking torque to the rear axle. Besides, the slip rates can be real-timely calculated by the hierarchical controller due to the fact that wheels speed can be calculated by the motors/generators' speed and the vehicle speed can be obtained by GPS instrument.

## 3. Hierarchical Control Strategy

### 3.1. The Flow Chart of the Control Strategy

Generally, the motors' speed can be obtained real-timely from the CAN bus, which is one of the advantages of the electric vehicles. The wheels speed can be calculated from the motors' speed without wheel speed sensors. It is worth noting that because of the vehicle speed which can be directly obtained by the GPS instrument, the slip rates of front and rear wheels can be easily calculated.

**Figure 2:**
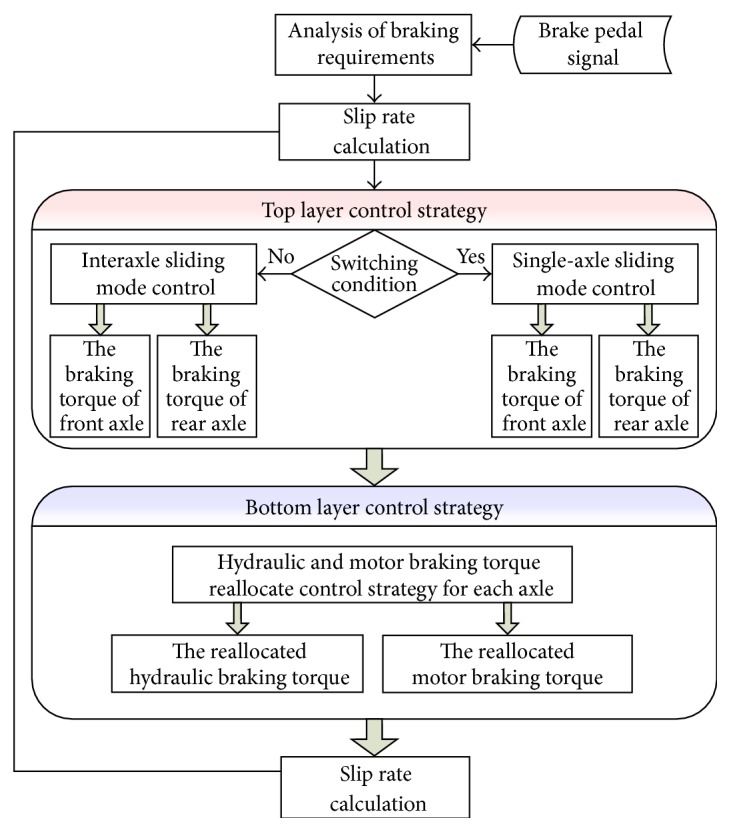
The hierarchical control strategy.

Figure 2 shows the hierarchical control strategy. Optimizing the braking stability is the main object of the top layer and maximizing the regenerative braking energy recovery efficiency is the main object of the bottom layer. Based on the switching condition, the top layer can choose a suitable control strategy to maintain the braking stability. If the interaxle sliding mode control strategy is worked, a braking severity signal is also needed, which can be got from the analysis of the driver's braking intention. On the contrary, if the single-axle sliding mode control strategy worked, the top layer can adjust the braking force distribution without the braking severity signal. After adjusting, the distributed braking torque will be delivered to the low layer. Under the control strategy of the bottom layer, the maximum generating torque which is subjected to the generators and battery will be calculated firstly, and, then, a reallocation control strategy will be worked to reallocate the distributed braking torque to the generators and hydraulic brakes.

### 3.2. The Top Layer

#### 3.2.1. The Switching Condition

The top layer needs to judge the switch condition real-timely to realize the strategy switch. Generally, if the slip rate is lower than *S*_opt_ which is the slip rate corresponding to the peak adhesion coefficient (about 15%~20%), the vehicle will stay in the general braking condition. On the contrary, the vehicle will be in emergency braking condition. The maximum adhesion coefficient is usually around *S*_opt_, which is also the optimum value for the cooperative braking system. However, for the single-axle sliding mode control, the target value is also *S*_opt_, which will lead the slip rate to be fluctuating around this value; namely, if the switch condition is *S*_opt_, both the control strategies will be switched repeatedly. So the switching condition is defined as

(1) $$\begin{array}{c} S_{f}>S_{shift}||S_{r}>S_{shift},\\ S_{shift}=S_{opt}-\delta_{s}, \end{array}$$

where *δ*_*s*_ is the relaxation factor and *S*_shift_ is the control mode shift threshold. In this paper, take *S*_opt_ = 20% and *δ*_*s*_ = 5%.

#### 3.2.2. The Interaxle Sliding Mode Strategy

According to the top layer in Figure 2, if both of the slip rates are lower than *S*_shift_, the general braking condition will be recognized. As stated in the introduction, the ideal braking force distribution strategy is good for the braking stability. However, it is also a risky control strategy with the variation of the loaded condition. So, how to realize a dynamic ideal braking force distribution strategy in the general braking condition is one of the key technologies in the top layer.

In this paper, an interaxle sliding mode strategy is proposed to equate the front and rear wheels' slip rates by adjusting the distribution of the braking force between front and rear wheels, which can guarantee the braking stability at general braking condition. The excellent adaptability to the parameter fluctuation is a highlighted advantage for the sliding mode control. Additionally, the slip rates of the wheels can always be real-timely calculated under any load case by the top layer. So the ideal braking force distribution strategy will always be obtained and will be unaffected by the varied loaded condition.

According to the definition of the slip rate, the interaxle sliding mode strategy can be realized by the identical wheel speed control. The switching function can be described as

(2) $$\begin{array}{c} s=\omega_{f}-\omega_{r}+c\overset{t}{\underset{0}{\int }}\left(\omega_{f}-\omega_{r}\right)dt, \end{array}$$

where *c* is the weighting parameter which denotes the slope value of the sliding curve and *ω*_*f*_ and *ω*_*r*_ are the rotation speed of the front and rear wheels, respectively.

Taking a derivative of (2), we can give

(3) $$\begin{array}{c} \dot{s}={\dot{\omega }}_{f}-{\dot{\omega }}_{r}+c\left(\omega_{f}-\omega_{r}\right), \end{array}$$

where $\dot{s}$ is the reaching law.

According to the dynamic equation of the vehicle, we have

(4) $$\begin{array}{c} {\dot{\omega }}_{f}=\frac{F_{f}r-T_{f}}{I},\\ {\dot{\omega }}_{r}=\frac{F_{r}r-T_{r}}{I}, \end{array}$$

where *T*_*f*_ and *T*_*r*_ denote the braking torque of the rear wheels, respectively, *F*_*f*_ and *F*_*r*_ denote the longitudinal braking tire force of the front and rear wheels, respectively, *I* is the moment of inertia of the wheels, and *r* is the radius of the wheels.

Substituting (4) into (3), we can get

(5) $$\begin{array}{c} T_{r}-T_{f}=\dot{s}I+F_{r}r-F_{f}r-cI\left(\omega_{f}-\omega_{r}\right). \end{array}$$

Generally, the reaching law is vital to accelerate the reaching process. In this paper, we adapt to the exponential reaching law:

(6) $$\begin{array}{c} \dot{s}=-\varepsilon sgns-ks,\;\varepsilon >0,\;\;k>0 \end{array}$$

(7) $$\begin{array}{c} s\left(t\right)=\left\{\begin{array}{cc} \frac{\varepsilon }{k}+\left(s_{0}-\frac{\varepsilon }{k}\right)e^{-kt}, & s>0\\ \frac{-\varepsilon }{k}+\left(s_{0}+\frac{\varepsilon }{k}\right)e^{-kt}, & s<0, \end{array}\right. \end{array}$$

where *s*_0_ is the initial value of *s*.

Substituting (6) into (5), another equation can be realized as

(8) $$\begin{array}{c} T_{r}-T_{f}=\left(-\varepsilon sgns-ks\right)I+F_{r}r-F_{f}r-cI\left(\omega_{f}-\omega_{r}\right). \end{array}$$

During the general braking, the total required braking torque of the vehicle can be obtained by the braking severity, which is the result of the analysis of the driver's braking intention:

(9) $$\begin{array}{c} T_{require}=\delta mgzr, \end{array}$$

where *T*_require_ is the total required braking torque of the vehicle, *m* is the vehicle mass, *g* is the acceleration of gravity, and *z* is the braking severity. *δ* is the rotary mass coefficient. In addition, *T*_require_ is also equal to the sum of *T*_*f*_ and *T*_*r*_:

(10) $$\begin{array}{c} T_{require}=T_{f}+T_{r}. \end{array}$$

Combining (5) with (10), we can get

(11) $$\begin{array}{c} T_{r}=\frac{\left(-\varepsilon sgns-ks\right)I+F_{r}r-F_{f}r+mgzr-cI\left(\omega_{f}-\omega_{r}\right)}{2},\\ T_{f}=\frac{mgzr+\left(\varepsilon sgns+ks\right)I-F_{r}r+F_{f}r+cI\left(\omega_{f}-\omega_{r}\right)}{2}. \end{array}$$

#### 3.2.3. The Single-Axle Sliding Mode Control Strategy

As shown in the switching condition, if any of the slip rates is bigger than *S*_shift_, the single-axle sliding mode control will be activated. According to the braking theory, if the slip rates of the tires fluctuated around *S*_opt_, the longitudinal adhesion ratio and the lateral adhesion ratio will be maximized simultaneously. So both the braking distance and the braking stability will be improved. In addition, similar to the interaxle sliding mode strategy, a good control strategy which can adapt to different road surfaces and load cases also takes a vital important role.

The switching function in this control strategy of the front wheel can be expressed as

(12) $$\begin{array}{c} s_{fi}=S_{f}-S_{opt}, \end{array}$$

where *S*_*f*_ is the slip rate of the front wheels, which is calculated as follows:

(13) $$\begin{array}{c} S_{f}=\frac{v-\omega_{f}r}{v}. \end{array}$$

Taking a derivative of (12) and (13), we get

(14) $$\begin{array}{c} {\dot{s}}_{fi}={\dot{S}}_{f}=\frac{-{\dot{\omega }}_{f}r+\left(1-S_{f}\right)\dot{v}}{v}. \end{array}$$

Combining (14) with (4), we get

(15) $$\begin{array}{c} T_{f}=\frac{Iv{\dot{s}}_{fi}}{r}+F_{f}r-\frac{I\left(1-S_{f}\right)\dot{v}}{r}. \end{array}$$

The reaching law of this sliding mode is the same as the interaxle sliding mode:

(16) $$\begin{array}{c} {\dot{s}}_{fi}=-\varepsilon sgns-ks,\;\varepsilon >0,\;\;k>0, \end{array}$$

where *s*(*t*) has the same description as (7).

Substituting (16) into (15), *T*_*f*_ can be rewritten as

(17) $$\begin{array}{c} T_{f}=\frac{Iv\left(-\varepsilon sgns-ks\right)}{r}+F_{f}r-\frac{I\left(1-S_{f}\right)\dot{v}}{r}. \end{array}$$

Similarly, *T*_*r*_ can be deduced as the same method of *T*_*f*_:

(18) $$\begin{array}{c} T_{r}=\frac{Iv\left(-\varepsilon sgns-ks\right)}{r}+F_{r}r-\frac{I\left(1-S_{r}\right)\dot{v}}{r}. \end{array}$$

### 3.3. The Bottom Layer

How to reallocate the distributed braking torques to the generators and hydraulic brakes to maximize the regenerative braking energy recovery is the core technology in this paper. Two technologies should be studied: the first one is the real-time calculation of the maximum charging torque and the other is the reallocation strategy between generators and hydraulic brakes.

#### 3.3.1. The Maximum Charging Torque Calculation

Generally, the maximum charging torque is affected by two factors. One is the maximum output generating torque of the generators, which is constrained by the given generator speed and the torque characteristics of motor. The other is the maximum input charging torque of the battery, which is restricted by the current state of charge (SoC).

The minimum of the two factors gives the maximum charging torque:

(19) $$\begin{array}{c} T_{charging}=min\left\{T_{generating},T_{recharging}\right\}, \end{array}$$

where *T*_charging_ is the maximum charging torque, *T*_generating_ is the maximum output generating torque of the generators, and *T*_recharging_ is the maximum input charging torque of the battery.

*T*_generating_ can be obtained as follows:

(20) $$\begin{array}{c} T_{generating}=T_{generating1}+T_{generating2}\\ T_{generating1}=f_{T1}\left(n_{1}\right)\\ T_{generating2}=f_{T2}\left(n_{2}\right), \end{array}$$

where *n*_1_, *n*_2_ denote the speeds of the generators 1 and 2, respectively, *T*_generating1_, *T*_generating2_ denote the maximum generating torque of generators 1 and 2, respectively, and *f*_*T*1_(*n*_1_), *f*_*T*2_(*n*_2_) denote the maximum generating torque which can be obtained by Figure 5 through interpolation method.

**Figure 3:**
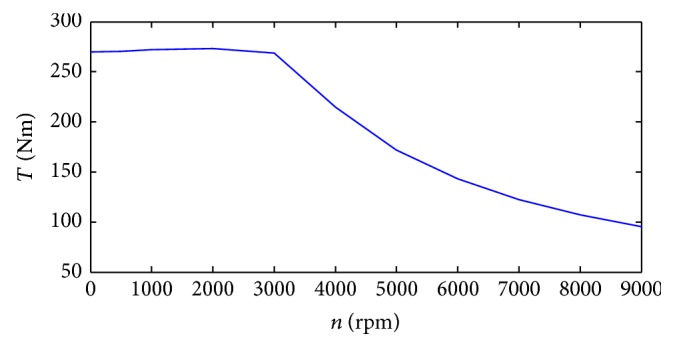
The generating torque versus speed curve.

Additionally, if the generator speed is lower than 500 rpm, the generating efficiency and the control precision will be limited; in this paper, we define that if *n* < 500 rpm, then make *T*_generating_ = 0 (Figure 3).

*T*_recharging_ can be calculated as follows.

Firstly, according to the current SoC, the charging power can be expressed as follows:

(21) $$\begin{array}{c} P_{recharging}=\left\{\begin{array}{cc} P_{recharging\_max} & SoC\leq 0.3\\ \frac{0.8-SoC}{0.5}P_{recharging\_max} & 0.3<SoC<0.8\\ 0 & SoC\geq 0.8, \end{array}\right. \end{array}$$

where *P*_recharging_max_ is the maximum charging power of the battery and *P*_recharging_ is the recharging power.

**Figure 4:**
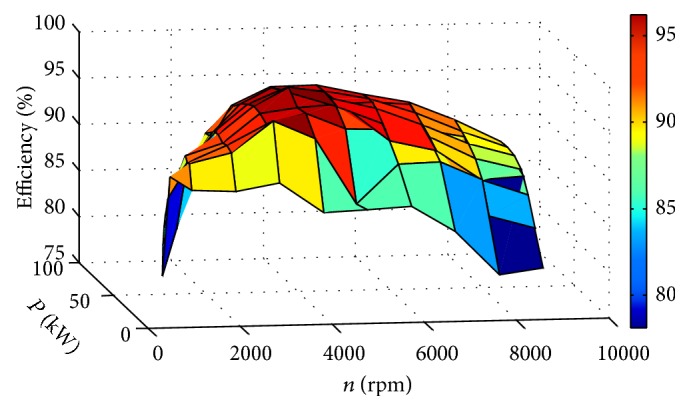
The efficiency map of the generators.

Then we can get

(22) $$\begin{array}{c} T_{recharging}=\frac{9550P_{recharging}}{n\eta \left(n,P_{recharging}\right)}, \end{array}$$

where *η*(*n*, *P*_recharging_) is the efficiency of the generator, which is subjected to the speed *n* and the recharging power *P*_recharging_, as shown in Figure 4.

#### 3.3.2. The Reallocation Strategy

**Figure 5:**
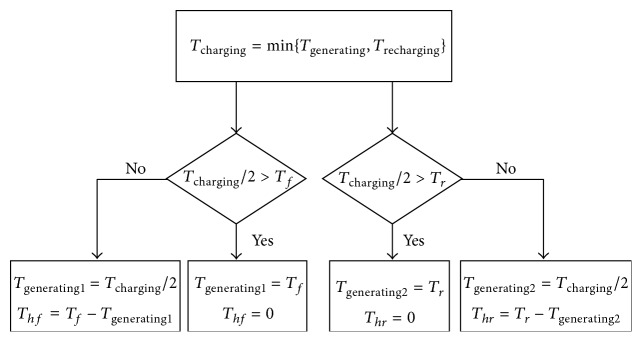
The reallocation strategy.

The basic principle for the bottom layer is to fulfill the maximum charging torque, for the sake of improving the regenerative braking energy recovery efficiency. It can be described as Figure 5 shows.

 With respect to the front axle
 if *T*_charging_/2 is bigger than *T*_*f*_, then make *T*_generating1_ = *T*_*f*_ and *T*_*hf*_ = 0. If *T*_charging_/2 is lower than or equal to *T*_*f*_, then make *T*_generating1_ = *T*_charging_/2, *T*_*hf*_ = *T*_*f*_ − *T*_generator1_.
 With respect to the rear axle
 if *T*_charging_/2 is bigger than *T*_*r*_, then make *T*_generating2_ = *T*_*r*_ and *T*_*hr*_ = 0. If *T*_charging_/2 is lower than or equal to *T*_*r*_, then make *T*_generating2_ = *T*_charging_/2, *T*_*hr*_ = *T*_*r*_ − *T*_generator2_,

where *T*_generator1_ and *T*_generator2_ denote the reallocated charging torques of generator 1 and generator 2, respectively, and *T*_*hf*_ and *T*_*hr*_ denote the reallocated braking torques of the front and rear hydraulic brakes, respectively.

## 4. Simulation Results and Discussion

Based on the MATLAB/Simulink software, a simulation model of the vehicle is set up. The initial vehicle speed is defined as 64.8 km/h, and the initial SoC is 0.5. Moreover, two load cases are provided to verify the strategy adaptability to parameter changes, which are shown in Table 1.

### 4.1. General Braking Condition

Generally speaking, the braking severity under a dry payment is mainly lower than 0.35, which will take up 95% proportion [18]. In this braking condition, the road surface is defined as dry payment and the peak adhesion coefficient is set as 0.8. To verify the interaxle sliding mode strategy, two braking cases are carried out, both of which are the mainly braking cases.

1. The braking severity is 0.15, which is defined as braking case 1.
2. The braking severity is 0.3, which is defined as braking case 2.

**Figure 6:**
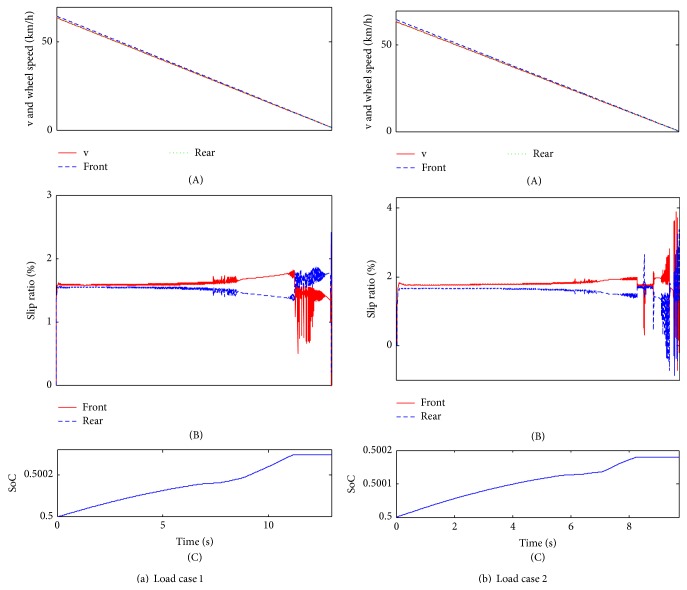
The simulation results of braking case 1.

As shown in Figure 6(a), the times taken for stopping the vehicle are approximately 13 s for load case 1 and 9.7 s for load case 2. Additionally, since the braking severity is relatively small, the wheel speeds can follow the vehicle speed well, which is the contribution of the interaxle sliding control strategy. At the same time, the slip rates between front and rear wheels of the two load cases are extremely close before the braking vehicle speed of 25 km/h (Figure 6(b)). It means that the vehicle can maintain the ideal braking force distribution mode under the control of the interaxle sliding control strategy. Subsequently, from the vehicle speed of about 25 km/h, the slip rates begin to be inconsistent; however, the inconsistency is small, and the most important is that the slip rate of the front wheel is bigger than the rear wheel, which can ensure the braking safety if the vehicle may be locked inevitably (the adhesion ratio of the front wheel is bigger than the rear wheel). At the end of the braking, the slip rates will be fluctuated remarkably, and the interaxle sliding mode control effectiveness is not obvious. The last and one of the most important performances is that SoC are increased to around 0.50021 for load case 1 and 0.50017 for load case 2. What is worth mentioning is that the bigger the vehicle mass is, the higher SoC will be. In addition, after the braking vehicle speed of 12.5 km/h, SoC will keep a constant value as a result of the bottom layer's control strategy which defined that if *n* < 500 rpm, make *T*_generating_ = 0.

**Figure 7:**
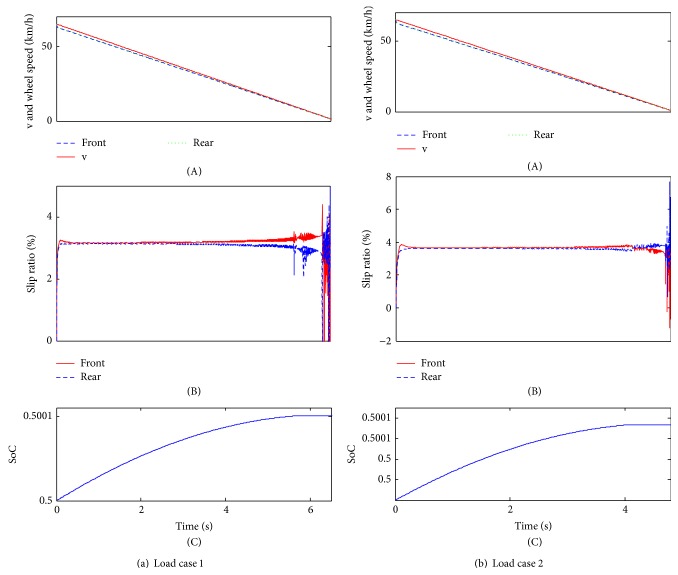
The simulation results of braking case 2.

The simulation results in Figure 7(a) show that the braking time is around 6.6 s for load case 1 and 4.8 s for load case 2. Both of the wheel speeds excellently follow the vehicle speed. Comparing to the slip rates between braking case 1 and braking case 2, the braking case 2 has a better control effectiveness. For load case 1, before the braking vehicle speed of 10 km/h, the slip rates are extremely close; for load case 2, only at the end of the braking deceleration do the slip rates have a little fluctuation. As a whole, the interaxle sliding mode strategy takes good control effeteness in this braking case. With respect to SoC, obviously, they will increase for both load cases: for load case 1, the SoC will increase to around 0.50008 and, for load case 2, the SoC will increase to around 0.500071. Comparing to the braking case 1, the increasing rate will be relatively small, which indicates that the higher the braking severity is, the lower SoC will be. The same as braking case 1, SoC will also keep a constant value after the braking vehicle speed of 12.5 km/h.

Given the above analysis, the interaxle sliding mode control strategy has a good adaption for different braking cases and load cases. At mainly braking cases, the braking force distribution between the front and rear wheels can reach the ideal distribution; at the same time, the regenerative braking energy recovery efficiency can be further improved.

### 4.2. Emergency Braking Condition

The single-axle sliding mode control strategy aims at keeping the slip rates at about 20%, by which the vehicle can keep great longitudinal force and lateral force simultaneously, thereby improving the braking stability performance. Particularly in the emergency braking condition, a good control strategy can avoid the wheels being locked before stopping. In addition, to verify the adaptability to different road surfaces, two road surfaces are also provided in Table 2. The road surfaces are represented by the peak adhesion coefficient at each wheel.

**Figure 8:**
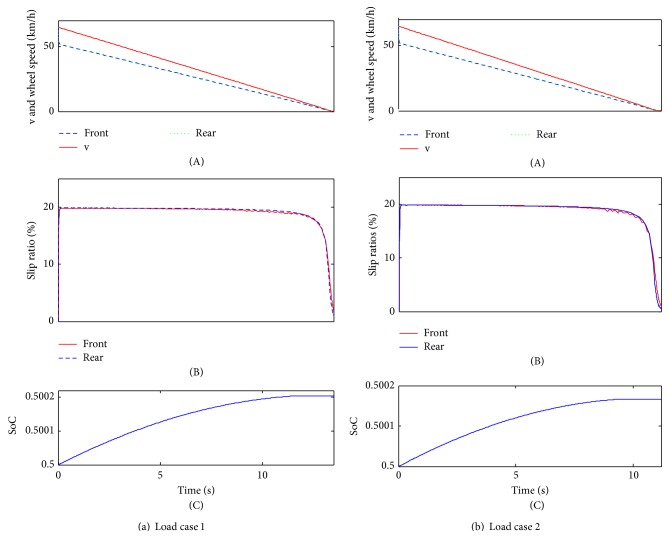
The simulation results of low and uniform load surface.

Figure 8(a) shows that the vehicle stops around 13.5 s for load case 1 and 11.3 s for load case 2 and both load cases will not be locked on the low and uniform road surface. During braking deceleration, the slip rates will be adapted to about 20%, which is the target value of the single-axle sliding mode control strategy. As a result, the vehicle can get the maximum lateral braking force and keep a better braking stability. At the end of braking, the slip rate will slow down sharply, due to the low braking vehicle speed (Figure 8(b)). With regard to SoC, load case 1 has increased to approximately 0.500203; load case 2 has increased to around 0.500166. Comparing to the simulation results of the load cases, the single-axle sliding mode control strategy has preferable adaptability to different load cases.

**Figure 9:**
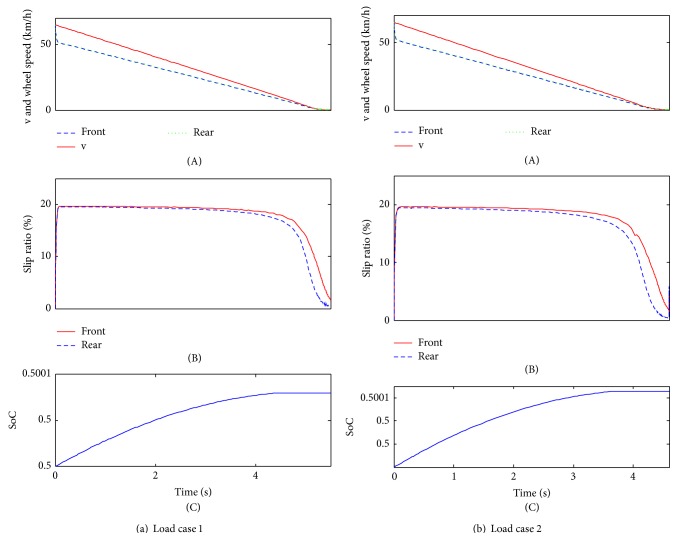
The simulation results of joint surface.

As shown in Figure 9(a), both load cases will not be locked under the joint road surface. For load case 1, the braking time is about 5.4 s; for load case 2, the braking time is around 4.6 s. The slip rate control effeteness is also very preferable for both of the load cases; at the end of braking, due to the low braking vehicle, the single-axle sliding mode control is not obvious (Figure 9(b)). Additionally, SoC will be increased to 0.5008 for load case 1 and 0.500066 for load case 2 (Figure 8(c)). Similarly, the adaptability is also very good for different load cases.

In view of the above analysis, the hierarchical control strategy can adapt to different road surfaces and different load cases. As a result, the vehicle can keep a preferable braking stability during emergency braking condition and improve the regenerative braking energy recovery efficiency.

### 4.3. Simulation Results When Passing the Complex Surfaces

**Figure 10:**
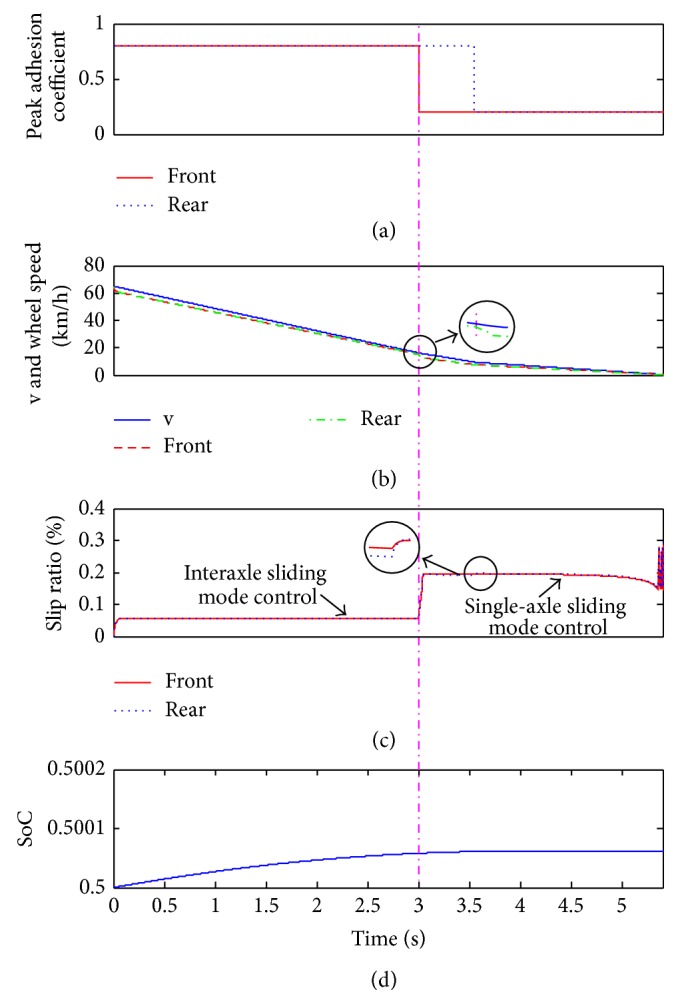
The simulation results when passing the complex surface.

As shown in Figure 10(a), the vehicle was designed to pass the pavement from high adhesion surface to the low adhesion surface. The peak adhesion coefficient of the higher side was 0.8, and the lower side was 0.2. On this condition, the vehicle will pass three kinds of typical road surfaces in turn, which are uniform and high adhesion surface, joint adhesion surface, and uniform and low adhesion surface, respectively. The braking severity was defined as 0.3, and load case 1 was used.

From the braking time of 0 to 3 s, the vehicle was braking on the uniform and high adhesion surface; the general braking condition was selected. As a result, the slip rate control was based on the interaxle sliding mode control strategy, and the slip rates between front and rear wheels were extremely close (Figure 10(c)); correspondingly, the wheel speeds will perfectly follow the vehicle speed (Figure 10(b)). In addition, SoC was increasing constantly with the braking process. Subsequently, the peak adhesion coefficient of the front axle declined rapidly to 0.2, and the rear axle was still on the high adhesion side. The slip rate of the front wheel will be increased sharply; once the slip rate meets the switching condition, the top layer will switch to the single-axle sliding mode control strategy; then, the control strategy will strive to keep the slip rates of the front and rear wheels around 20%. It is worth noting that the wheel speeds will decrease suddenly at the switching point, and shortly after that the wheel speed will preferably follow the vehicle speed again. Besides, SoC was always increasing during this braking time until the speed of the generator was less than 500 rpm.

## 5. Conclusions

A hierarchical control strategy, including the interaxle sliding mode control strategy for the general braking condition, the single-axle sliding mode control strategy for the emergency braking condition, and the maximum charging torque strategy for the maximum regenerative braking energy recovery efficiency were studied in this paper.

The simulation results under the general braking condition show that the interaxle sliding mode control strategy could adapt to different load cases and keep the slip rates between front and rear almost consistence. The bottom layer could improve the regenerative braking energy recovery efficiency.

Aiming at the emergency braking condition, two typical road surfaces were also proposed and verified through the single-axle sliding mode control strategy. The simulation results indicate the slip rates were preferably controlled to about 20% for different emergency braking cases and different load cases. Moreover, the regenerative braking energy recovery efficiency was also improved.

A simulation was also carried out to verify the vehicle braking from one road surface to another, which proves that the hierarchical control strategy has a strong adaptability to different types of surfaces.

The hardware in the loop experiment for hierarchical control strategy and the vehicle road test will be considered in our next step work.

## Figures and Tables

**Table 1:** The load cases.

	Mass (kg)	The front wheel base (m)	The rear wheel base (m)	Centroid height (m)
Load case 1	5000	1.479	1.071	0.603
Load case 2	3550	1.483	0.996	0.569

**Table 2:** The road surfaces.

Typical road surface	Adhesion coefficients	
	The front wheel	The rear wheel
Low and uniform	0.2	0.2
Joint	0.2	0.8
